# What are the drivers of waiting times, waiting lists and backlog during and following the COVID-19 pandemic?

**DOI:** 10.1093/eurpub/ckac129.211

**Published:** 2022-10-25

**Authors:** L Siciliani

**Affiliations:** Department of Economics and Related Studies, University of York, York, UK

## Abstract

The COVID-19 pandemic put a halt to the number of patients being treated thus generating larger excess demand and a greater mismatch between demand for health care services and the supply of services provided. This presentation will provide a conceptual framework for understanding the dynamic interrelation between waiting times, waiting lists and the backlog over time. Data from different countries will be used to illustrate and rationalise how waiting times, lists and volumes evolved over time. It will then discuss factors driving the demand and supply of care during Covid, and emphasise the critical role of supply in absorbing the backlog and reducing the waiting list under different scenarios, as well as factors on the demand side both in the short run and the long run. Supply determinants include the availability of health workers as key factor to “bounce back”, their productivity and provider capacity (hospital beds, operating theatres), the cost of providing treatment in a safe environment, financial capacity to fund additional supply both by public and private providers, interventions to minimise staff exhaustion and burnout, payment systems which are aligned with higher volumes, and technologies and digital solutions. Demand determinants include ageing and rising chronic conditions, and multi-morbidity patients (including long-COVID patients), increasing expectations, new technologies, prioritisation protocols, but also fear of infection which can leading to a temporary or permanent reduction in demand but an increase in unmet need. The framework will be used to discuss policy options both on the demand and the supply side to deal with the backlog, but also to improve the resilience and efficiency of health systems.

